# Intravenous and Subcutaneous Pharmacokinetic Modeling to Support the Development of Long-Acting Multipurpose Prevention Technology for HIV and Pregnancy

**DOI:** 10.3390/biomedicines14040873

**Published:** 2026-04-10

**Authors:** Nathan Engel, Daniel Oliveira, Craig Sykes, Amanda P. Schauer, Jasmine L. King, Thy Le, Soumya Rahima Benhabbour, Mackenzie Cottrell

**Affiliations:** 1 Division of Pharmacotherapy and Experimental Therapeutics, UNC Eshelman School of Pharmacy, University of North Carolina at Chapel Hill, 301 Pharmacy Ln, Chapel Hill, NC 27599, USAcraig_sykes@unc.edu (C.S.);; 2 Lampe Joint Department of Biomedical Engineering, North Carolina State University, 4130 Engineering Building III, Raleigh, NC 27695, USAbenhabs@email.unc.edu (S.R.B.); 3 Lampe Joint Department of Biomedical Engineering, University of North Carolina at Chapel Hill, 10010 Mary Ellen Jones, Chapel Hill, NC 27599, USA; 4 Division of Pharmacoengineering and Molecular Pharmaceutics, UNC Eshelman School of Pharmacy, University of North Carolina at Chapel Hill, 301 Pharmacy Ln, Chapel Hill, NC 27599, USA

**Keywords:** HIV PrEP, cabotegravir, long-acting, pharmacokinetics, multipurpose prevention technologies, ISFI, MPA

## Abstract

**Background/Objectives**: Women and girls, particularly in sub-Saharan Africa, face high risks for both HIV and unintended pregnancy. Inconsistent condom use underscores the need for new multipurpose prevention technologies (MPTs) that combine HIV pre-exposure prophylaxis (PrEP) and contraception. Long-acting (LA) injectables are especially promising. To this end, an LA cabotegravir (CAB)/medroxyprogesterone acetate (MPA) in situ-forming implant (ISFI) has been developed. We report pharmacokinetic (PK) modeling to characterize CAB and MPA disposition and absorption to support the development of the MPT ISFI. **Methods**: Female BALB/c mice received single intravenous (IV) or subcutaneous (SQ) bolus doses of CAB or MPA. Sparse plasma samples were collected (~3 mice/timepoint) for PK analysis by LC-MS/MS. Noncompartmental analysis assessed SQ bioavailability. Macroparameterized compartmental PK models were fit to IV data to derive unit impulse responses (UIRs) for each drug. **Results**: CAB and MPA exhibited 61% and 42% bioavailability, respectively. CAB IV PK was best described by a two-compartment model with macroconstant parameters: A = 16,621 ng/mL, α = 4.52 h^−1^, B = 30,206 ng/mL, and β = 0.053 h^−1^. MPA IV PK was also best described by a two-compartment model, with A = 2506 ng/mL, α = 10.5 h^−1^, B = 439 ng/mL, and β = 0.65 h^−1^. These values define the UIR for CAB and MPA. **Conclusions**: Our IV PK modeling framework fully characterizes CAB/MPA disposition in mouse, enabling downstream deconvolution-based estimation of absorption from controlled-release formulations. This provides a foundation for in vitro–in vivo correlation, facilitating preclinical evaluation of long-acting formulations such as ISFIs.

## 1. Introduction

In 2024, women and girls made up 45% of new HIV infections diagnosed globally and 63% of those diagnosed in sub-Saharan Africa [1]. Another challenge women and girls face, especially in low- and middle-income countries, is unintended pregnancies. A study of girls and young women in Uganda found a 23.5% incidence of unintended pregnancy, with higher risk present among those who had multiple sexual partners in recent months and those who reported not using contraceptives [2]. Having multiple concurrent sexual partners is a shared risk factor for HIV acquisition [3], highlighting the intersection of HIV and unintended pregnancy as a significant global health concern.

There are currently four FDA-approved HIV pre-exposure prophylaxis (PrEP) options available, including two daily oral regimens and two long-acting (LA) injectable regimens [4]. Of the approved LA regimens, cabotegravir-LA (CAB-LA) is a bimonthly 3 mL intramuscular injection that demonstrated 79% risk reduction compared to daily oral PrEP. This improved effectiveness among clinical trial participants was attributed to reduced adherence burden of a provider-administered injection compared to a daily self-administered pill [5]. Of concern, CAB-LA is non-removable, and it continues to release suboptimal but detectable CAB concentrations for up to 15 months after the first missed injection [6]. This could select for integrase strand transfer inhibitor resistance in someone who acquires HIV after discontinuing PrEP or falling out of care [5]. Additionally, CAB-LA requires a total of 6 clinic visits a year, which is uniquely challenging in low- and middle-income countries. More recently, lenacapavir was approved as a twice-yearly LA injectable PrEP for adults and adolescents, where it demonstrated 100% protection with superiority over daily oral tenofovir-based PrEP in cisgender women in South Africa and Uganda [6]. This level of protection was associated with high (92.8%) adherence in participants receiving lenacapavir at week 52, while participants receiving a tenofovir product showed 7–11% adherence at week 52. However, the limitations of lenacapavir PrEP include a lack of reversibility and nodule formation at injection sites.

Unlike HIV PrEP, there is a wide range of contraceptive options available, such as daily oral pills, implants, intrauterine devices, condoms, and injections. Depot medroxyprogesterone (DMPA, DepoProvera^®^) is the only approved injectable contraceptive, but it displays similar limitations to CAB-LA, such as it being non-removable and non-biodegradable and having prolonged concentrations that delay fertility for a median of 9 months after discontinuation [7]. Of the various contraceptive methods, condoms are the only approved product that can prevent both HIV acquisition and unintended pregnancy. However, inconsistent and incorrect condom use hinders the effectiveness of the prevention of HIV, sexually transmitted diseases, and unintended pregnancy [8,9]. One viable solution to this challenge is multipurpose prevention technologies (MPTs), which are being developed to provide dual protection against HIV and unintended pregnancies by combining agents with unique therapeutic indications (such as an antiretroviral for HIV prevention and a hormonal contraceptive for the prevention of unintended pregnancies) into a single product. They are being developed in forms such as pills, gels, rings, implants, and injectables, with LA injectables being particularly appealing for their convenience, privacy, and low user burden [10]. However, to our knowledge, no injectable MPTs are reported to be in clinical development.

In situ-forming implants (ISFIs) are biodegradable injectables made from a biodegradable polymer and biocompatible water-miscible solvent. After subcutaneous injection, solid depots form via phase inversion that release drug over extended periods [11]. Previous work by Young et al. demonstrates that long-acting MPT ISFIs are capable of delivering CAB and MPA in BALB/c mice [12]. This drug delivery system was well tolerated, and CAB plasma concentrations were maintained above the 4PA-IC90 pharmacokinetic (PK) benchmark of 660 ng/mL [13] over 90 days, while also displaying sustained, detectable MPA plasma concentrations throughout the 90-day timeframe [12], acting as preliminary preclinical evidence for the efficacy of CAB/MPA ISFIs for dual protection against HIV and contraception. Of note, this long-acting MPT ISFI may overcome the adherence limitations associated with daily oral PrEP and condom use, while also addressing the limitations of CAB-LA or DMPA by offering removability.

The results of the long-acting MPT ISFI in Young et al. are promising and warrant further evaluation in other relevant animal models. However, the individual characterization of the systemic drug disposition of CAB and MPA, as well as bioavailability from the dosing compartment, is a prerequisite for subsequent formulations studies. Because plasma concentrations observed from subcutaneously administered implants reflect the combined influence of subcutaneous bioavailability and drug release from the implant (absorption), as well as systemic disposition (distribution and clearance), this poses a challenge in determining the true absorption profile from implant PK data alone, which is vital for clinical translation. Therefore, analysis of SQ and intravenous (IV) PK following immediate-release drug solutions is typically needed to understand drug loss due to incomplete bioavailability and capture systemic disposition free from any absorption processes [14]. Accordingly, we do not evaluate MPT ISFI formulations in this study. Instead, we characterize IV and subcutaneous (SQ) PK in mice using simple bolus solutions to isolate systemic disposition and estimate bioavailability. We fit macroparameterized compartmental PK models to derive the unit impulse response (UIR) for CAB and MPA. These UIRs establish a foundation for the future deconvolution of ISFI absorption profiles in subsequent studies, with the ultimate purpose of guiding future development for clinical translation.

## 2. Materials and Methods

### 2.1. In Vivo Pharmacokinetics of BALB/c Mice

All experiments were carried out with a protocol approved by the University of North Carolina Animal Care and Use Committee. Female BALB/c mice (8–10 weeks old; Jackson Laboratory, Ban Harbor, ME, USA) were used to obtain in vivo pharmacokinetic profiles of CAB and MPA from both SQ and IV injection. Injections were immediate release bolus solutions of CAB and MPA. Separate groups of mice were used for each compound and route of administration. SQ CAB (60 µg) and SQ MPA (60 µg) were administered using unique animals per group, and IV CAB (66 µg) and IV (65 µg) MPA were also administered using unique animals per group. IV samples for CAB and MPA were collected at 0.08, 1, 3, 6, 8, 18, 24, and 30 h. These IV timepoints plus 48 and 72 h were collected for SQ MPA, and the IV timepoints plus 120, 168, 336, 504, and 720 h were collected for SQ CAB. Plasma was isolated by placing blood in capillary tubes coated with EDTA. Blood was processed to plasma by centrifugation and then stored at −80 °C until pharmacokinetic analysis was conducted. We sought to minimize the effect of potential confounders by randomizing animals to each treatment group and ensuring that cages of mice in different treatment groups were housed within the same environmental conditions within the facility.

### 2.2. Bioanalytical Methods

Mouse CAB and MPA plasma concentrations were extracted with liquid–liquid extraction using isotopically labeled internal standards (13C, 2H2, 15N-CAB, MPA-d6). Reverse-phase chromatography with a water/acetonitrile gradient containing 0.1% formic acid was used for separation. Drug quantification was performed on an AB Sciex API-5000 triple quadruple mass spectrometer under positive ion electrospray conditions for analyte and internal standard detection. The lower limits of quantification were 25.0 ng/mL and 0.500 ng/mL for CAB and MPA, respectively. All calibrators and quality control samples were within 15% of the nominal value for both within-day and between-day runs in accordance with established regulatory guidelines [15]. Raw quantified CAB and MPA plasma concentration data for both SQ and IV can be found in the Supplementary Materials (File S1).

### 2.3. Pharmacokinetic Modeling

IV and SQ PK data were analyzed to separate systemic disposition from absorption. This approach supports future deconvolution analyses by allowing for the estimation of the true absorption profile in SQ ISFIs free from confounding from flip-flop PK [14]. Concentrations below limits of quantification (BLQ) were imputed at ½ the lower limit of quantification (LLOQ) according to Beal M6 [16]. The first BLQ values were imputed, with subsequent BLQ values dropped from the analysis. Noncompartmental analysis (NCA) was conducted for SQ and IV datasets of CAB and MPA using Phoenix WinNonLin v8.5 (Certara, Inc., Princeton, NJ, USA). CAB IV and SQ concentrations in the terminal elimination phase were averaged, while MPA IV and SQ concentrations used raw data for subsequent calculations. Potential outliers were evaluated with Grubbs’ test (two-sided; α = 0.05), and any significant outliers were excluded prior to model development. The terminal elimination rate constant (λ_z_) informed the calculation of AUC_0-inf_, and bioavailability was calculated as the ratio of dose-normalized SQ to IV AUC_0-inf_. The linear-log trapezoidal method was used for AUC calculation.

Population PK modeling was performed using CAB and MPA IV PK data in Phoenix NLME v8.5 (Certara USA Inc., Princeton, NJ, USA). Structural model selection was guided by the naïve pooled algorithm to determine the appropriate model with one, two, and three compartments using macroconstant parameterization. Model selection was based on objective function and Akaike Information Criterion (AIC) values, parameter precision, condition number, and goodness-of-fit diagnostics. Subsequent population modeling employed traditional maximum likelihood or expectation maximization estimation algorithms, with the latter being potentially advantageous for sparse sampling [17]. Random effects were incorporated when supported by parameter precision and acceptable shrinkage. Initial parameter estimates were informed by NCA results, where terminal slope intercept (λ_zint_) was used for B, initial concentration (C_0_) − λ_zint_ was used for A, and regression slopes were fit to early and late samples for α and β, respectively. The final estimated fixed-effect parameters included A, α, B, and β, and the A and B (ng/mL) parameters were normalized to the absolute doses of CAB and MPA (66 µg and 65 µg, respectively). Both models were validated via nonparametric bootstrap with 250 resamples and visual predictive check (VPC) with 250 simulated datasets. All simulations, bootstrap analyses, and VPCs were performed in Phoenix.

## 3. Results

A total of n = 23 plasma samples were collected for IV CAB. One mouse that was scheduled for a 30 h collection timepoint died following injection. Additionally, one 24 h concentration was removed as an outlier. A total of n = 24 plasma samples were collected for IV MPA. All samples collected after 8 h were BLQ. A total of n = 45 plasma samples were collected for SQ CAB. All samples collected after 120 h were BLQ. A total of n = 30 plasma samples were collected for SQ MPA. All samples collected after 18 h were BLQ.

**Figure 1:**
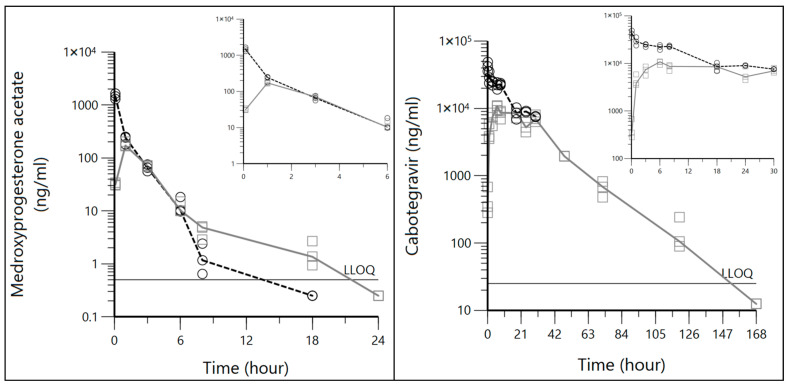
Median medroxyprogesterone acetate (MPA) and cabotegravir (CAB) plasma concentration vs. time plots. Intravenous (black dashed line) and subcutaneous (gray solid line) data are shown along with the lower limit of quantification (LLOQ) for each drug. The LLOQ was 0.500 ng/mL for MPA and 25.0 ng/mL for CAB. Each circle or square represents a single sample or mouse. Outlier and non-imputed BLQ data are omitted.

The observed IV and SQ plasma concentration time data for CAB and MPA are reported in Figure 1. MPA exhibits comparable elimination rates and concentration levels across both routes of administration from 1 to 6 h, with slightly sustained concentrations after 8 h for the SQ route. CAB exhibits comparable concentrations at 18 and 30 h across both routes of administration. The time to maximum concentration (Tmax) is noticeably delayed for SQ CAB (6 h) relative to SQ MPA (1 h).

Noncompartmental analysis results are reported as mean estimates for CAB and MPA (where applicable) by IV or SQ administration in Table 1. SQ bioavailability (%F) was estimated as 61% for CAB and 42% for MPA.

The final fixed-effect parameter estimates of the MPA and CAB IV macroconstant models are reported in Table 2, alongside nonparametric bootstrap means. The coefficient of variation (CV%) is included for both model estimates, as well as the 95% CI for bootstrap mean estimates. There is good agreement between model estimates and their bootstrap means for both MPA and CAB, suggesting reasonable estimates. Additionally, parameter-estimated CV%s are low for both MPA and CAB, indicating high precision.

The final CAB IV macroconstant model was best described with power residual error and without random effects for α, and parameter estimation was performed using the QRPEM algorithm. The final MPA IV macroconstant model was best described with log additive error without random effects for A and β, and parameter estimation was performed using the FOCE ELS method.

**Figure 2:**
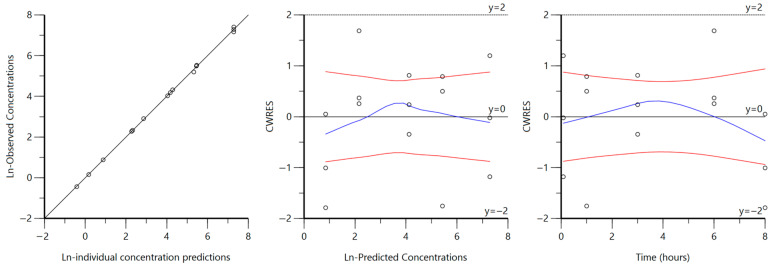
MPA IV macroconstant model goodness-of-fit plots. Ln-transformed observed vs. predicted concentration (**left**) is shown with the line of unity. Conditional weighted residuals vs. ln-transformed predicted concentration (**middle**) and conditional weighted residuals vs. time (**right**) are shown with loess regression lines. Blue lines show deviation from the 0-reference line and the red lines show negative reflection. All concentrations are in units of ng/mL.

**Figure 3:**
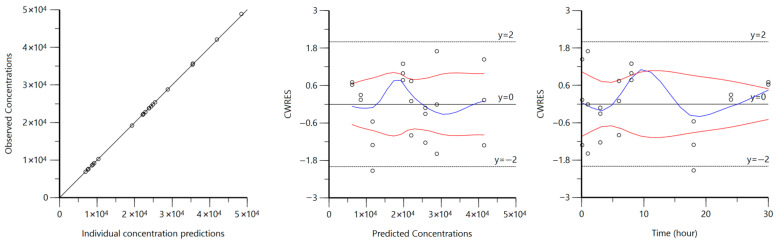
CAB IV macroconstant model goodness-of-fit plots. Ln-transformed observed vs. predicted concentration (**left**) is shown with the line of unity. Conditional weighted residuals vs. ln-transformed predicted concentration (**middle**) and conditional weighted residuals vs. time (**right**) are shown with loess regression lines. Blue lines show deviation from the 0-reference line and the red lines show negative reflection. All concentrations are in units of ng/mL.

Goodness-of-fit diagnostic plots for intravenous macroconstant models are displayed in Figure 2 and Figure 3 for MPA and CAB, respectively. All observed concentrations fall along the line of unity in the observed vs. predicted concentration plot for both MPA and CAB. MPA residual plots show reasonable homoscedasticity, although CAB residual plots show some heteroscedasticity around a predicted concentration of around 20,000 ng/mL and 8 h.

**Figure 4:**
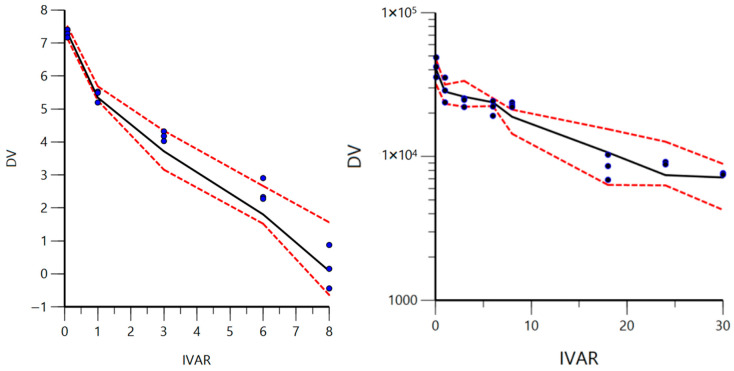
Visual predictive checks for final IV macroconstant models of MPA (**left**) and CAB (**right**) using n = 250 simulated datasets. DV represents concentration in ng/mL (ln transformed in MPA) while IVAR represents time in hours. Observed concentrations are shown as blue dots with BLQ values omitted. Red dotted lines indicate the 5th and 95th predicted quantiles while the solid black line indicates the median predicted concentrations.

Visual predictive checks were used to further validate our IV macroconstant models and are shown in Figure 4. Most of the observed MPA concentrations fall within the range of the 5th and 95th predicted quantiles through the 8 h time course. Similarly, most of the observed CAB concentrations fall withing the 5th and 95th predicted quantiles through the 30 h time course.

## 4. Discussion

Here, we fit macroparameterized compartmental PK models to derive the UIRs for CAB and MPA for downstream applications of deconvolving the subcutaneous absorption profile for MPT ISFIs under development. We also demonstrate incomplete subcutaneous bioavailability within the mouse model (61% and 42% for CAB and MPA, respectively). Based on our final macro-model fits, a biexponential UIR would be required to deconvolve long-acting CAB and MPA PK data with the following form (Equation (1)):

(1) $$UIR\left(t\right)=\frac{A}{Dose}e^{-\alpha t}+\frac{B}{Dose}e^{-\beta t}$$

Applying our final estimates and respective doses, the UIRs for CAB and MPA are shown in Equation (2) and Equation (3), respectively, in units of ng/mL/ng. Time (t) is in units of hours.

(2) $$UIR_{CAB}\left(t\right)=0.252e^{-4.52t}+0.458e^{-0.053t}$$

(3) $$UIR_{MPA}\left(t\right)=0.0386e^{-10.5t}+0.00675e^{-0.65t}$$

These UIRs can be used to obtain the deconvolved concentration time absorption profiles for each drug.

We utilized a sparse sampling design, where three mice were sampled per timepoint, with each mouse contributing a single plasma sample. This approach poses a limitation for our population pharmacokinetic analysis, as the estimated random effects did not reflect true interindividual variability. Although random effect parameter estimates are not explicitly reported, population PK modeling was performed within a mixed-effects framework because the inclusion of random effects minimizes the bias of fixed-effect parameter estimates relative to a naïve pooled analysis [18]. Fixed-effect parameters were of primary interest as they are sufficient to characterize the mean absorption and disposition of CAB/MPA to define the UIRs. The search for, or inclusion of, the covariate effects on PK parameters was impractical due to sparse sampling and for our deconvolution purposes.

We imputed the first timepoint that exhibited concentrations BLQ as ½ LLOQ and omitted the remaining values. The percentage of each dataset that was imputed was 0%, 17%, 8%, and 14% for IV CAB, IV MPA, SQ CAB, and SQ MPA, respectively. This has the potential to introduce bias into our NCA and model parameter estimation, but because we are confident that omitted BLQ concentrations are indeed decreasing through time, and because the relative percentage of imputed data was low (<10% on average), this method was appropriate for our purposes.

Our NCA results demonstrate two different measures of AUC, but we ultimately opted to use AUC_0-inf_ for bioavailability assessment to better capture the entirety of drug exposure. The percentage extrapolated AUC_0-inf_ for these calculations was low (0.1%, 0.2%, 25%, and 0.09% for MPA IV, MPA SQ, CAB IV, and CAB SQ, respectively). We pre-specified ≤20% AUC, extrapolated to be reliable, but 25% for IV CAB was considered acceptable given the sparse sampling design and long terminal half-life of CAB in mice. In the macroconstant modeling for CAB, the QRPEM algorithm was chosen, as expectation-maximization methods are often better equipped for parameter estimation in sparse data compared to maximum likelihood methods [17]. For MPA macroconstant modeling, however, FOCE ELS performed well and was thus chosen. Weight -normalized dosing did not affect macroconstant model fit, likely due to low animal weight variability.

The final model selection was supported by various criteria. Two compartment models best described the observed IV data for both CAB and MPA, which is consistent with the biexponential decay seen in early timepoints in Figure 1. Although bootstrap analyses were limited to 250 resamples, this was considered adequate given the small dataset (n = 21 for CAB; n = 18 for MPA); the results supported the robustness of the parameter estimates (Table 2). Interestingly, greatest parameter imprecision (high estimate CV% and wide bootstrap 95% CI) was found for A for both CAB and MPA, suggesting greater variability in early drug concentrations governed by rapid distribution phase kinetics. This is not unexpected, as other parameters are better informed by datapoints covering greater spans of time. Additionally, high precision of the β estimate for MPA suggests that our M6 imputation method was robust.

Diagnostic plots (Figure 2 and Figure 3) also generally support our final chosen models, with any observed heteroscedasticity likely attributed to the sparse sampling rather than model misspecification. Furthermore, VPCs for both models demonstrated model adequacy (Figure 4) as the majority of observed concentrations fell between the predicted 5th–95th quantiles. A common trend in both VPCs was the presence of tighter predicted intervals at early timepoints, followed by wider intervals at later timepoints, reflecting relative uncertainty associated with greater or fewer plasma samples observed, respectively. The CAB VPC showed a few observed concentrations outside of the predicted interval at 8 h, which corresponds to the previously noted heteroscedasticity in the residual plots. While this suggests a limited ability to accurately characterize or predict concentrations at the 8 h time point for the CAB model, the overall disposition is well described, with sparse sampling likely driving time-specific deviations. Together, bootstrap analysis, parameter precision, goodness-of-fit plots, and VPCs demonstrate that the final macroconstant model parameter estimates are reliable for use in deconvolution.

Together, these findings show that IV PK modeling can reliably characterize systemic disposition of CAB and MPA. These models provide the foundational UIRs to deconvolve the SQ absorption profile in MPT ISFI formulations to estimate the true absorption profile in future studies. Future applications of the UIR-based deconvolution framework will enable estimation of the rate and extent of drug absorption, as well as the prediction of the residual drug in the implant, which can be experimentally validated. This validated framework can then be used to characterize in vivo absorption kinetics and quantify the remaining drug over time in additional relevant animal models, thus supporting dose and regimen considerations in the development of CAB/MPA ISFIs as a multipurpose prevention technology.

## Figures and Tables

**Table 1:** Noncompartmental analysis results.

Parameter	Mean MPA Estimates		Mean CAB Estimates	
	IV Bolus	SQ Bolus	IV Bolus	SQ Bolus
Dose (μg)	65	60	66	60
T_1/2_ (h)	3.95	2.88	14.2	16.2
C_0h_ (ng/mL)	1743	N/A	43,656	N/A
C_max_ (ng/mL)	1470	180.7	42,233	10,383
T_max_ (h)	N/A	1	N/A	6
AUC_partial_ (ng·h/mL)	1334	488.5	463,668	222,475
AUC_0-inf_ (ng·h/mL)	1344	524.9	619,264	342,132
AUC_0-inf_/Dose (ng·h/mL/ng)	0.0207	0.00874	9.382	5.702
AUC_0-inf_ %Extrapolated	0.1	0.2	25	0.09
%F	N/A	42	N/A	61

AUC_partial_ was defined as AUC_0–8h_ for MPA and AUC_0–30h_ for CAB. %F was calculated based on dose-normalized AUC_0-inf_. The presence of N/A values for C_0h_, T_max_, and %F are due to the irrelevance of the given parameter in the context of a specific route of administration.

**Table 2:** Macroconstant parameter and bootstrap estimates for intravenous medroxyprogesterone acetate (MPA) and cabotegravir (CAB).

Parameter	MPA Estimates		MPA Bootstrap (n = 250)		CAB Estimates		CAB Bootstrap (n = 250)	
	Mean	CV%	Mean	95% CI	Mean	CV%	Mean	95% CI
A (ng/mL)	2506	21	2274	(1519, 3303)	16,621	29	17,799	(6256, 26,985)
B (ng/mL)	439	11	451	(335, 602)	30,206	4.8	30,018	(27,083, 33,328)
α (h^−1^)	10.5	23	9.25	(3.85, 13.6)	4.5	5.4	4.5	(4.0, 5.0)
β (h^−1^)	0.65	6.3	0.66	(0.58, 0.75)	0.053	11	0.052	(0.040, 0.066)

## Data Availability

Data will be archived according to FAIR standards within an SQL database that is being developed as a publicly accessible PK data sharing service through the NIH award U24AI181685. Currently, data can be made available through this data sharing service by specific request.

## Supplementary Materials

The following supporting information can be downloaded at: https://www.mdpi.com/article/10.3390/biomedicines14040873/s1, File S1: Raw quantified CAB and MPA plasma concentration data.
